# The Power of Patient Engagement With Electronic Health Records as Research Participants

**DOI:** 10.2196/39145

**Published:** 2022-07-08

**Authors:** Jeff Pawelek, Katie Baca-Motes, Jay A Pandit, Benjamin B Berk, Edward Ramos

**Affiliations:** 1 Digital Trials Center Scripps Research Translational Institute La Jolla, CA United States; 2 CareEvolution Ann Arbor, MI United States

**Keywords:** electronic health record, EHR, digital health technology, digital clinical trial, underrepresentation, underrepresented in biomedical research, biomedical research

## Abstract

Electronic health record (EHR) technology has become a central digital health tool throughout health care. EHR systems are responsible for a growing number of vital functions for hospitals and providers. More recently, patient-facing EHR tools are allowing patients to interact with their EHR and connect external sources of health data, such as wearable fitness trackers, personal genomics, and outside health services, to it. As patients become more engaged with their EHR, the volume and variety of digital health information will serve an increasingly useful role in health care and health research. Particularly due to the COVID-19 pandemic, the ability for the biomedical research community to pivot to fully remote research, driven largely by EHR data capture and other digital health tools, is an exciting development that can significantly reduce burden on study participants, improve diversity in clinical research, and equip researchers with more robust clinical data. In this viewpoint, we describe how patient engagement with EHR technology is poised to advance the digital clinical trial space, an innovative research model that is uniquely accessible and inclusive for study participants.

## Introduction

The electronic health record (EHR) represents an evolution from static paper-based records to a more portable, interactive, and dynamic medium shared by tools like web-based patient portals and mobile apps. The wealth of information provided in each EHR—such as medical history, medications, diagnoses, treatments, procedures, allergies, laboratory tests, immunizations, hospital admissions, and clinic visits—creates new opportunities. Advancements in how patients access their EHR have resulted in significant expansion in how EHR data are operationalized by providers to inform and deliver care. Further, the patient’s ability to access and share their EHR data directly with researchers has opened the door for the research community to glean clinically important information from study cohorts.

The mechanisms by which patients interact with their EHR are in a fluid state of development, and patients expect improved functionality in how they interact with their EHR [1]. It is no surprise that the expanding reach of the digital health system for ambulatory data capture coincides with the expansion of digital clinical research that seeks to leverage these data to gain insights at both individual and population levels. As digital health technologies become more accessible and integrated into daily living, the increasing ubiquity of real-world and real-time health data stands to transform how researchers address questions about health and disease. In this viewpoint, we describe how recent advancements in EHR technology are advancing the digital clinical trial space, an innovative research model that is uniquely accessible, scalable, inclusive, and impactful for study participants and researchers.

## Policy Feeding Progress

Over a decade ago, the Health Information Technology for Economic and Clinical Health (HITECH) Act was enacted to incentivize the “meaningful use” of health information with an emphasis on more widespread adoption of EHRs by hospital systems and medical practices. While there is evidence that the HITECH Act stimulated the uptake of EHR systems across more medical facilities, the development of an interoperable health IT environment using modern internet technology and technical standards was not adequately addressed [2-4]. As a result, subsequent small- and large-scale initiatives have helped unlock some of the more advanced capabilities of EHR connectivity and compatibility.

Harvard Medical School and Boston Children’s Hospital created Substitutable Medical Apps and Reusable Technology (SMART), an application programming interface standard that establishes compatibility to allow any EHR-based software application to function with any EHR system, thus equipping hospitals with a broader selection of EHR tools to support ever-changing clinical and business needs. However, the introduction of Fast Healthcare Interoperability Resources (FHIR) proved to be a tipping point and a crucial piece to the elaborate puzzle of health IT infrastructure. The not-for-profit organization Health Level Seven International (HL7) created FHIR, a technical standard that defines how EHR data are accessed and exchanged between different computer systems. Given HL7’s robust global community of developers and stakeholders, the FHIR standard gained significant traction within the health IT community [5]. The coupling of the SMART and FHIR standards (known as “SMART on FHIR”) is now considered an essential toolkit by hospitals, researchers, and the health IT industry to improve the interoperability of EHR systems [6,7].

**Figure 1:**
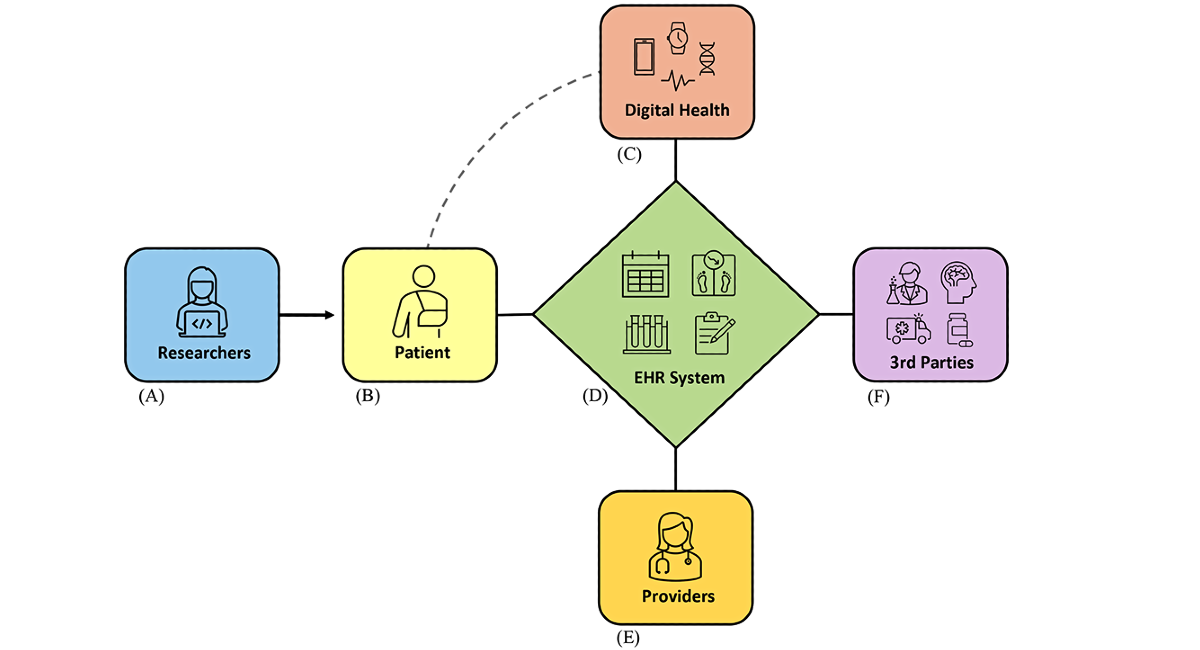
The widespread adoption of SMART on FHIR technical standards has enabled EHR systems to serve as a hub for the secure and efficient exchange of digital health information. (A) Researchers can partner directly with patients to participate in clinical research, and patients can chose to grant permission to researchers to access and use their EHR data; (B) patients can view and manage their EHR through an online patient portal using mobile and desktop devices; (C) patients can link their personal digital health products (eg, fitness trackers, wearable health monitors, at-home genomic tests) to their EHR as a way to centralize various elements of their health information; (D) EHR systems allow patients to schedule appointments with their provider, view provider notes, communicate with their provider, complete routine health surveys, and find opportunities to participate in research; (E) providers enter their clinical notes into their patient’s EHR, access external patient-provided digital health information, and work with their patient to ensure critical health information is accurate and current; (F) patients can link their health information from third-party services such as outside providers, imaging centers, laboratories, and pharmacies. EHR: electronic health record; FHIR: Fast Healthcare Interoperability Resources; SMART: Substitutable Medical Apps and Reusable Technology.

More recently, SMART on FHIR resources have been integrated with Epic, Cerner, and other widely used EHR platforms to operationalize clinical decision support tools for providers, such as risk prediction tools for surgical procedures and advanced treatment modalities [8,9]. SMART on FHIR was recently used by Apple for their Health app, which allows users to link their device-generated health data directly to their EHR as a means to consolidate their digital health information [10]. The latest federal mandate, the 21st Century Cures Act, primarily relies on FHIR to expand meaningful *patient use* of EHR systems by incentivizing advancements to patient-facing digital health services and apps, bidirectional sharing of health information between patients and providers, and patient-mediated sharing of EHR with researchers [11,12]. These recent developments have equipped patients with the ability to use their mobile or desktop devices to access physician’s notes and laboratory test results, schedule medical appointments, link health and activity monitors, and search for and enroll in clinical trials (Figure 1).

## The Expanding Role of EHR in Clinical Research

The COVID-19 pandemic put a spotlight on digital health and whether existing technologies were poised to face the unique challenges of a global health crisis forcing remote patient monitoring. Perhaps the most rapid and expansive implementation of digital health during the pandemic was the shift to telehealth, which demonstrated that basic digital technologies were adequate to support the widespread delivery of virtual health care [13]. Other EHR-driven solutions included the ability for patients to create advance care plans, in case of severe illness; new templates to capture COVID-19 test results to inform population-level statistics; and predictive models to stratify risk and inform clinical decision-making for infected patients [14-16]. The integration of these digital health tools—driven in part by large-scale exchange and compilation of patient data—emphasizes the unique role EHR technology plays in addressing complex health problems. For clinical research, increasing EHR adoption provides evermore data to complement health survey and wearable device data, supplement missing data, and reduce participant burden.

The digital nature of EHRs makes them well-suited to be incorporated into decentralized clinical research. In contrast to the traditional, hospital-based paradigm of clinical research, decentralized studies utilize a siteless, patient-centered model that affords study participants the convenience of remote data acquisition (both active and passive) through a combination of mobile apps, wearable devices, electronic surveys, self-collected biosamples, and now—with the advancements described above—patient-mediated EHR connectivity. While the “digital divide” and other barriers still exist, decentralized digital clinical trials can be accessible to a broader spectrum of patient populations—including those who are underrepresented in biomedical research (UBR)—and diminish common barriers and selection biases such as health insurance status, medical provider affiliation, and proximity to an academic medical center [17]. Direct-to-participant recruitment strategies equip researchers with multiple avenues to engage a large pool of potential study participants, thus increasing the sample size and statistical power with which site-based research chronically struggles. Additionally, local institutional review boards offer researchers who are not affiliated with a covered entity (eg, academic medical center) more expedient regulatory pathways to study approval and initiation since HIPAA (Health Insurance Portability and Accountability Act) Privacy Rules are not applicable to noncovered entities [18].

## Patient-Mediated EHR Sharing in a Real-World Digital Clinical Trial

In response to the COVID-19 pandemic, the Scripps Digital Trials Center launched the Digital Engagement & Tracking for Early Control & Treatment (DETECT) digital research platform (ClinicalTrial.gov identifier: NCT04336020) [19,20]. DETECT is an observational research effort examining whether individualized changes in heart rate, activity, or sleep—all monitored through the use of a wearable activity tracker—can serve as early indicators of viral infections. DETECT leveraged a remote, siteless research model, and the participant experience allowed for a lightweight entry process. DETECT did not require participants to connect their EHR; however, the study app allowed participants the option to share their EHR data to equip the researchers with additional information for post hoc analyses. Among the entire DETECT cohort (n=40,322), approximately 10% (n=4210) elected to connect their EHR within the study app (enrollment numbers as of March 21, 2022).

DETECT’s foundational protocol was designed to support additional substudies aimed at more specific clinical questions. DETECT-At Home Early Alert and Diagnosis (DETECT-AHEAD) is one such substudy that explores the feasibility of an algorithm-driven notification system based on data from wearable sensors, with a specific focus on outcomes from UBR populations. Study participants receive an alert via the study app to perform an at-home COVID-19 test, possibly before they experience symptoms, to rule out infection as soon as possible. For DETECT-AHEAD, sharing of EHR data is a criterion for participation, so all study participants connected their EHR after completing eligibility surveys. The protocol design for DETECT-AHEAD also set goals for enrollment of UBR populations to ensure the substudy cohort was reflective of the US population (Table 1).

In DETECT-AHEAD, the average age of participants was 49.4 years, the male-to-female ratio was 0.68, 15.3% (n=69) of the cohort was under 35 years of age, and racial minorities comprised 30.4% (n=137) of the cohort. DETECT-AHEAD demonstrates that while it is possible to engage a diverse population of participants, more work needs to be done to reach UBR populations that are considered disadvantaged by the digital divide (ie, age ≥65 years, highest education grade <12, annual household income <$10,000) [21]. The methods were performed in accordance with relevant guidelines and regulations and approved by the Scripps Institutional Review Board. All study participants signed an electronic informed consent form.

**Table 1:** DETECT-AHEAD^a^ enrollment numbers in the underrepresented in biomedical research category (self-reported).

Characteristic		Participants (N=450), n (%)
Age (≥65 years)		71 (15.8)
Gender (other)		7 (1.6)
**Race**		
	Hispanic/Latino	45 (10)
	Asian	39 (8.7)
	Other (non-White)	27 (6)
	Black/African American	26 (5.8)
Highest level of education (grades 1-11)		2 (0.4)
Annual household income (<$10,000)		13 (2.9)

^a^DETECT-AHEAD: Digital Engagement & Tracking for Early Control & Treatment – At Home Early Alert and Diagnosis.

## Current Challenges and Limitations

While DETECT and DETECT-AHEAD demonstrate how study participants, including those from UBR groups, possess a willingness to share their EHR in a research setting, the research community continues to overcome hurdles to promote the adoption of patient-mediated EHR exchange mechanisms. Perhaps the most notable constraints are the longstanding disparities in universal access to reliable internet service and use of mobile technologies [22,23]. While the digital clinical trial model offers individuals more convenient ways to take part in research and thus fosters inclusivity, its reliance on broadband internet service continues to be a barrier.

Over a quarter (28%) of US adults who live in rural areas do not have broadband internet service, which may partly explain why rural communities interact less with EHRs compared to their urban counterparts [22,24]. Also, individuals who are 65 years and older are less likely to use EHRs, and while smartphone ownership has risen in this group in recent years, only 42% report owning a smartphone (compared to 77% of all adults over 18 years) [23,25]. African American, Asian, and Latino race, younger age (ie, <35 years), and low education level are other factors associated with low engagement with EHR systems [26]. However, there have been some positive trends in recent years. Nearly 60% of patients were offered an EHR patient portal by their health care provider—a 17% increase from 2014 to 2020 [27]. Additionally, the number of patients who downloaded their EHR data nearly doubled between 2017 and 2020, and roughly 20% of EHR users elected to link their health data to an outside caregiver, health service, or app [27]. Without improved access to the internet and connected digital health tools among UBR groups, clinical study outcomes will continue to lack diversity and thus have limited applicability.

The robustness of a patient’s EHR is critically important to both providers and researchers to ensure reliable interpretation and analysis of health information. Patients who actively manage their EHR can help ensure their information is up-to-date and free of errors, but many EHR patient portals still do not offer patients editing permission to allow full control over their own health information. From a technical standpoint, as capabilities to aggregate and access data across different sources increase, so does the challenge to integrate data from multiple modalities, deal with missing data, and map discrepant terminology, including data in free-text form. The EHR itself must continue to evolve and expand its capability, for example, to enrich the clinical context for data such as images or lab results. Additionally, the persistent concern for privacy and data security must also not be overlooked as we seek to find new ways of verifying identity, securely transferring EHR data, and improving deidentification techniques. Lastly, if an EHR system lacks quality control and safeguards against erroneous information, including improper or fraudulent use of the system, serious problems can arise such as diminished quality of care and medical errors [28].

## Moving Forward

As more health care organizations offer patients personalized tools to interact with and visualize their EHR data, patients will ultimately become more engaged with their provider and overall health management. Health IT professionals with expertise in user experience and interface design will serve increasingly important roles in optimizing patient engagement with their EHR and associated digital health tools. Equipping patients with permission to update information, correct errors, and connect external sources of health information is a critical step toward improving patient engagement with their EHR, which should become a universal feature across all EHR systems.

There are some promising technical solutions on the horizon. Increasingly, software services are available for cloud-based clinical data warehousing, entity extraction, terminology standardization, and record linkage, which leverage functionality developed by others at scale, obviating the need to solve these challenges for each application [29-32].

Accessing and sharing EHR data are not the only obstacles to recognizing the full potential of leveraging EHR data. Ideally, the EHR is more than a historical record of clinical outcomes but rather a dynamic asset in preventative interventions. However, for this to be realized, the EHR must continue toward a comprehensive capture of a patient’s health information to meaningfully provide information back to the participant by way of at-risk assessments, prediction of outcomes, or personalized detection of disease.

## Conclusion

EHR technology has made significant advances through improved compatibility across connected mobile devices, digital health products, and health IT software. The biomedical research community is beginning to harness the benefits of EHR connectedness by means of fully remote digital clinical trials, which help reduce burden on study participants and fosters diversity and inclusivity of study populations. As more patients become familiar with their EHR to manage their ever-growing sources of health information, engage with their provider, and partner with researchers, the health care community as a whole will be better equipped to optimize health and well-being for all.

## Abbreviations

DETECT: Digital Engagement & Tracking for Early Control & Treatment
DETECT-AHEAD: Digital Engagement & Tracking for Early Control & Treatment – At-Home Early Alert and Diagnosis
EHR: electronic health record
FHIR: Fast Healthcare Interoperability Resources
HIPAA: Health Insurance Portability and Accountability Act
HITECH: Health Information Technology for Economic and Clinical Health
HL7: Health Level Seven International
SMART: Substitutable Medical Apps and Reusable Technology
UBR: underrepresented in biomedical research

## Data Availability

All interested investigators will be allowed access to the analysis data set after approval of a proposal by a responsible authority at Scripps and with a data access agreement pledging to not reidentify individuals or share the data with a third party. All data inquiries should be initially addressed to the corresponding author.
